# Evaluating Disparities in Proton Radiation Therapy Use in AHOD1331, a Contemporary Children's Oncology Group Trial for Advanced-Stage Hodgkin Lymphoma

**DOI:** 10.14338/IJPT-21-00012.1

**Published:** 2021-10-28

**Authors:** Raymond B. Mailhot Vega, Sharon M. Castellino, Qinglin Pei, Susan Parsons, Kenneth B. Roberts, David Hodgson, Anne-Marie Charpentier, Thomas J. Fitzgerald, Sandy K. Kessel, Frank G. Keller, Kara Kelly, Bradford S. Hoppe

**Affiliations:** 1 Department of Radiation Oncology, University of Florida, Jacksonville, FL, USA; 2 Department of Pediatrics, Emory University/Aflac Cancer and Blood Disorders Center, Children's Healthcare of Atlanta, Atlanta, GA USA; 3 Department of Biostatistics, Children's Oncology Group, Statistics and Data Center, University of Florida, Gainesville, FL, USA; 4 Institute for Clinical Research and Health Policy Studies and Tufts Cancer Center, Tufts Medical Center, Boston, MA, USA; 5 Department of Radiation Oncology, Yale University School of Medicine, Yale Cancer Center, New Haven, Connecticut, USA; 6 Department of Radiation Oncology, Princess Margaret Cancer Center and University of Toronto, Toronto, Ontario, Canada; 7 Department of Radiation Oncology, Centre hospitalier de l'Université de Montréal, Montreal, Quebec, Canada; 8 Imaging and Radiation Oncology Core (IROC), Rhode Island, Lincoln, RI, USA; 9 Department of Radiation Oncology, University of Massachusetts Medical School, Worcester, MA, USA; 10 Department of Pediatrics, Emory University School of Medicine, Atlanta, GA, USA; 11 Department of Pediatrics, Roswell Park Comprehensive Cancer Center; University at Buffalo Jacobs School of Medicine and Biomedical Sciences, Buffalo, NY, USA; 12 Department of Radiation Oncology, Mayo Clinic Florida, Jacksonville FL, USA

**Keywords:** particle therapy, proton therapy, radiation therapy, health disparities, Hodgkin lymphoma

To the Editor:

The indications for proton radiation therapy carry the strongest evidence in pediatric cancers. In a recently published letter, Bitterman et al [1] reviewed factors associated with receipt of proton radiation therapy in patients enrolled in Children's Oncology Group (COG) solid tumor and CNS tumor trials. They demonstrated that Black children were less likely to receive this treatment than non-Hispanic white patients, a disparity that persisted when controlling for other demographic and clinical variables. We strongly commend them for their work, as addressing racism and infrastructural barriers to care requires its identification.

Disparities and unequal access to radiation therapy reflect barriers and systemic biases of the health system. As radiation oncologists are highly specialized, it is rare for them to primarily diagnose cancers, particularly in the pediatric setting. Unequal access reflects those obstacles or biases acting on (1) the relationship between pediatric oncologist and radiation oncologist; (2) the recommendation of radiation oncologist; or (3) patient choice. Determining what factors and processes lead to disparities as evidenced previously are paramount in addressing them.

The aforementioned analysis did not include lymphoma, as it focused on pediatric solid tumors. Furthermore, the authors characterized a cohort generally enrolled more than a decade ago. Proton therapy represents a radiation modality with the potential to reduce dose to healthy tissues. Within the context of Hodgkin lymphoma, especially for patients with large mediastinal adenopathy (LMA), dose reduction is important for at-risk organs, including the lungs, heart, and breasts [2, 3]. We now share our analysis of factors associated with proton therapy receipt on a contemporary COG trial for Hodgkin lymphoma, AHOD 1331 [4]. The primary aim of the randomized clinical trial was to determine the efficacy of brentuximab vedotin in a pediatric chemotherapy regimen for de novo high-risk classical Hodgkin lymphoma. Radiation therapy was indicated for all patients with LMA or slow-responding lesions after 2 cycles of chemotherapy on either treatment arm. As the primary trial endpoint has not been reported, we focus the analysis on factors associated with receipt of proton radiation therapy for LMA in Hodgkin lymphoma.

We conducted a univariate analysis by χ^2^ test to evaluate the association of receipt of proton radiation therapy with age (< or ≥12 years), sex, race, ethnicity, payment method as private versus nonprivate insurance, and neighborhood socioeconomic status (SES), based on a binary 5-digit zip code–derived census-tract definition of poverty level as low or non-low (< or ≥20%).

Among 265 patients with LMA who received radiation therapy, 65 (25%) received proton therapy and 200 (75%) received photon radiation therapy. The results of the analyses are presented in **Table 1**.

**Table 1.** Univariate analysis of radiation therapy receipt with demographic variables.

**Variable**	**All patients**	**Photon patients, n (%)**	**Proton patients, n (%)**	***P*** **value, χ^2^ test**
No. of patients	265	200	65	—
Age, mean ± SD, y	15.1 ± 3.0	15.1 ± 2.8	15.0 ± 3.6	.81
Age				.56
<12 y	39	28 (14.0)	11 (16.9)	
≥12 y	226	172 (86.0)	54 (83.1)	
Sex				.60
Female	146	112 (56.0)	34 (52.3)	
Male	119	88 (44.0)	31 (47.7)	
Race				.82
Black	33	24 (12.0)	9 (13.8)	
White	198	152 (76.0)	46 (70.8)	
Other	12	8 (4.0)	4 (6.1)	
Unknown	22	16 (8)	6 (9.2)	
Ethnicity				.81
Hispanic	55	39 (19.5)	16 (24.6)	
Non-Hispanic	199	152 (76.0)	47 (72.3)	
Unknown	11	9 (4.5)	2 (3.0)	
Payment method				.016
Nonprivate insurance	120	99 (29.0)	21 (26.2)	
Private insurance	145	101 (50.5)	44 (67.7)	
SES				.06
Low	29	20 (10.0)	9 (13.8)	
Non-low	214	159 (79.5)	55 (84.6)	
Missing	22	21 (10.5)	1 (1.5)	

**Abbreviation:** SES, socioeconomic status.

Among those receiving proton versus photon radiation therapy, 13.8% vs 12.0% were Black, 24.6% vs 19.5% were Hispanic, and 13.8% vs 10.0% were of low SES, respectively, with no difference in radiation therapy receipt by race, ethnicity, or SES detected. When payment method was evaluated, a significantly higher proportion of patients receiving proton radiation therapy were privately insured (67.7% with proton versus 50.5% with photon radiation therapy; *P* = .016).

Disparities in Hodgkin lymphoma outcome faced by Black children are documented [5, 6]. Black children with Hodgkin lymphoma have been shown to have significantly worse overall survival than their white counterparts in a large national database, and in children enrolled in COG trials. In contrast to the finding of Bitterman et al [1] of racial disparities in receipt of proton therapy, our analysis in a more contemporary national study for a hematologic cancer did not demonstrate racial or ethnic disparity in proton therapy access for children enrolled in trial. Given the different periods under which enrollment was conducted, the fact that more proton therapy centers existed during accrual for AHOD 1331, which enrolled from 2015 to 2019, may partly account for our findings compared to the historical trials included by Bitterman et al [1]. As our initial analysis is limited to the group with LMA, it is possible that the examination of disparities may yield different results when the entire radiation cohort is considered. With greater sample size and availability to analyze all enrolled children after the primary endpoint is released, it will be important to conduct multivariable analyses to control for any confounding factors to better evaluate individual associations. Given the ability of proton delivery to dosimetrically spare healthy organs and with favorable cost-effectiveness for survivors of Hodgkin lymphoma [7], proton therapy's allocation and receipt merits continued monitoring to ensure equal access.

The American Society of Clinical Oncology has called for continued prospective reevaluation of disparities in access to care and outcomes in order to eliminate inequalities that systemic and infrastructural racism may engender [8]. This is especially important with the advent of novel therapies like proton therapy with its goal of reducing second cancers and cardiotoxicity in survivors.
